# Flexibility and freedom suit me better: food delivery couriers’ preferred employment status

**DOI:** 10.3389/fsoc.2024.1415852

**Published:** 2024-06-17

**Authors:** Henri Kervola, Soili Hyvönen, Erika Kallionpää, Heikki Liimatainen

**Affiliations:** ^1^Transport Research Centre Verne, Tampere University, Tampere, Finland; ^2^School of Technology, JAMK University of Applied Sciences, Jyväskylä, Finland; ^3^School of Business and Economics, University of Jyväskylä, Jyväskylä, Finland

**Keywords:** courier, crowdshipping, employment status, food delivery, gig work, on-demand delivery, platform work, social responsibility

## Abstract

This research examines food delivery couriers’ preferred employment status and factors explaining their opinions. Previous studies have used qualitative research methods and are unable to explain couriers’ general views on employment status. In this research, a survey of 1,539 Wolt couriers was carried out in Finland with logistic regression, cross-tabulation, and content analysis as analysis methods. The results show that 56% of the couriers wanted to work as self-employed and 25% as employed. The opinion was most strongly explained by valuing work-related freedom and flexibility, which were associated with the right to refuse delivery tasks offered and to choose the amount of work, working hours and delivery vehicle. The preference for self-employment was also increased by the duration of courier work, one’s own choice to work as a courier, and age. Freedom and flexibility are dependent on the sufficient availability of delivery tasks, posing challenges when the demand is low.

## 1 Introduction

The key challenge in platform work is the unclear employment status of platform workers (Bilić and Smokvina, 2022). At the core of the problem is the question of whether platform workers should be self-employed or employed. More than 100 court decisions were issued in the EU regarding the professional status of people working through platforms. In one of the most recent court decisions, the Regional Administrative Court of Finland ruled that Wolt’s delivery couriers are self-employed (Harjumaa, 2024). In the EU alone, more than 28 million platform workers are affected, and according to estimates, the number of platform workers will increase to 43 million by 2025 (European Council, 2024). The European Commission has sought to improve the situation by adopting a directive in March 2024, according to which the degree of supervision and control exercised by platform companies is crucial in determining the employment status of workers. In the coming years, the Directive will be incorporated into the national legislation of the member states (Council of the European Union, 2024). However, at present the situation is ambiguous.

Platform work, also referred to as gig work, is defined as the matching of the demand for and supply of paid work through an online platform using an algorithm (Eurofound, 2021). Wood et al. (2019) divide gig work into local and remote gig work. Food delivery is a part of local gig work, and Wolt is one of the companies providing this service. They manage the transportation of customers’ orders from restaurants, grocery stores and retail stores and maintain their own grocery stores under the Wolt Market brand. Food and product distributors are usually called couriers (Moncef and Monnet Dupuy, 2021). Courier work is described as non-threshold work, which is well-suited for those who value freedom and want additional income (Kervola et al., 2022). In 2022, 3.9% of the Finnish population between 15 and 64 years worked in platform economy, of whom 26% sold items on platforms, 20% were content creators, 18% did courier work and the rest did apartment renting, taxi services and others (Pärnänen, 2023).

The employment status of couriers as self-employed workers has been critiqued by many researchers studying industrial relations in the context of platform work. However, there are only a handful of studies that concentrate on the employment vs. self-employment debate (Ahsan, 2020; Barratt et al., 2020; Vieira, 2021; Çiğdem, 2022; Lee, 2022). Earlier research gives some explanations on why couriers prefer self-employment such as the desire for freedom and flexibility but, on the other hand, finds background factors such as unemployment (Çiğdem, 2022) or immigrant status (Barratt et al., 2020) that seem to push them towards self-employment. Both Wood and Lehdonvirta (2021) and Maffie (2023) argue that platform companies use their power to influence workers’ perspectives on employment status. Some scholars argue that the freedom experienced by self-employed platform workers is in reality no freedom at all, but companies use it as a marketing tool to attract couriers in poor societal or economic positions (Popan, 2021).

There is a need to bring forward the broader courier point of view about how they perceive their employment status to grasp the motivations behind the debate. Vieira (2021), for example, has studied Spanish food delivery couriers’ willingness to work as self-employed through qualitative interviews, but this research seems to be an exception. Nevertheless, broader quantitative research is needed to understand the courier’s point of view on the issue since the generalizability of results is possible only with higher representability of the results. The purpose of this study is to fill this research gap.

The aim of this article is to understand and explain the motivations behind couriers’ preferred employment status. Three research questions were formed for this purpose.

1. Which employment status do food delivery couriers prefer? When couriers were given extra information in the survey about employment status, how did it change their preference?
2. What work related factors explain couriers’ willingness to be self-employed or work as employees?
3. What do couriers value the most as self-employed and as employees?

The second section of the article includes a literature review, followed by a discussion on the data and methods. The research results are introduced in the fourth section, and the fifth section discusses the findings. The sixth section concludes the article with the main findings and recommendations for future research.

## 2 Literature review

### 2.1 The change of work and the platform worker experience

Work on platforms represents a broader change in the overall labor environment. At the end of the 20th century, there has been a shift towards shorter and more unstable employment relations. The concept of a boundaryless career has emerged, which makes workers “contractors of choice” with discontinuous career paths that move beyond the boundaries of a single organization (Strauss et al., 2012). Van Doorn (2017) sees the change as part of the neo-liberalization of the economy, which is empowered by digitalization, and, as a result, treats the workforce as a service. Platform work has also increased in transport services, which can be divided into services for food and product delivery and taxi services (Szmelter-Jarosz and Rześny-Cieplińska, 2019). In food and product delivery, ordinary people carry out deliveries from restaurants, groceries, retail stores or warehouses to customer destinations using their own vehicles (Alnaggar et al., 2019), shared mobility systems or public transport (Serafini et al., 2018). Food and product delivery is also referred to as crowdshipping and crowdsourced delivery (Buldeo Rai et al., 2017).

Huws (2016) describes work on platforms as “logged” labor because the work is very much standardized, there is constant surveillance and monitoring of workers, and the worker is connected to an online platform in order to work. Platform work has been seen as a return to a Tayloristic mindset of people management, especially in the form of worker control. Algorithmic management is seen as a crucial characteristic of platforms, which means that the work is organized and guided by computer applications. Jarrahi et al. (2021, p. 2) define algorithmic management as a sociotechnical concept that is “emerging from the continuous interaction of organizational members and algorithmic systems.” It means that there is a constant negotiation over the way algorithms are used and, on the other hand, on the algorithm shaping the organizational roles and the power relations (Jarrahi et al., 2021). Vallas and Schor et al. (2020) see platforms representing a new type of economic activity as “permissive potentates,” which delegate control to participants but, on the other hand, keep the power to themselves. Companies’ power is manifested in avoiding direct employment of workers, allowing workers to enter into open employment relations, offering some supervision but omitting formal rules and creating spatial dispersion among workers (Vallas and Schor, 2020).

Algorithmic management has received plenty of criticism. One of the most studied platform companies, the taxi operator Uber, has been reported to exercise tracking and evaluation of workers using the algorithm to make automated decisions with low transparency and the workers being left without interaction with humans (Möhlmann and Zalmanson, 2018). Reid-Musson et al. (2020) highlight the drivers’ experiences of UberPool using punitive tactics that cause dissatisfaction among drivers and therefore their expectations of gig work are not met. Woodcock and Cant (2022) report on food delivery workers’ strikes, union organization and legal campaigns in Great Britain against Deliveroo. However, Wood et al. (2019) claim that the control exercised by algorithms deviates significantly from Taylorism in granting the worker significant levels of autonomy, task variety and complexity added with both spatial and temporal flexibility. Moreover, Joyce et al. (2023) argue that algorithmic control has been overemphasized in the recent literature. They point out that practices such as worker rating or nontransparent payment systems have been in place since the beginning of the capitalist work organization (Joyce et al., 2023). Thus, it seems that platform companies exercise power over workers, which does not necessarily represent a new organization of work but grants workers certain freedoms that are not usual in regular work contexts.

Gig workers also have positive experiences related to their work on platforms. Josserand and Kaine (2019) express that drivers in Australia appreciate many aspects of gig work, and Duggan et al. (2021) state that both food-delivery workers and taxi drivers described varying levels of engagement and satisfaction, but that the work seems to offer only limited advancement opportunities. Kusk and Bossen (2022) report the positive experiences of food delivery couriers working for Wolt in Denmark, who find the algorithm to be lenient and the payment system transparent and who receive support from a human support team. In their study, Mäntymäki et al. (2019) state that from the Uber and Lyft drivers’ perspective, flexibility in work relationships is the key positive element of platform-enabled work.

Schor et al. (2020) have argued that platform dependency influences the satisfaction levels of platform workers, especially towards their income. Those who are less dependent on the income the platform offers are more satisfied, while the dependent workers trying to sustain their lives through platform income are less satisfied (Schor et al., 2020). Glavin and Schieman (2022) have pointed out that the dependency also influences platform workers’ mental health, while the flexibility offered by platform work does not have a positive impact on the mental health of workers. Thus, the experience of workers seems complex towards platforms.

### 2.2 Platforms and self-employment

Most food delivery couriers are self-employed, which means that they work as sole proprietors or utilize other forms of entrepreneurship. There are some companies that use the employment model, such as Foodora used to do in the German market (Ivanova et al., 2018). Stewart et al. (2020) state that the line between employer control and market dictates is not clear especially when platform algorithms are able to reflect and predict market demand. Thus, determining, whether workers should be employees of self-employed, has remained unclear. Self-employment is usually promoted as autonomous work in which the nature of work can be determined independently, and the worker can enjoy flexibility (Ivanova et al., 2018; Wood et al., 2019). According to Çiğdem (2022), flexibility and freedom are often mentioned as the most important factors for a person choosing gig work. Another explanation that Martin (2016, p. 153) gives to workers preferring self-employment is that micro-entrepreneurship in the sharing economy promotes ‘individual economic empowerment’. Vieira (2021) recognizes both sides, freedom related to the use of time and income as the most important factors why Spanish platform workers protested against the Spanish government that wanted to make them employees. The entrepreneurship discussion is thus promoted as a form of self-development and an opportunity to make a choice to determine one’s own life (Gregory, 2021).

There are two explanations that Barratt et al. (2020) provide for the entrepreneurial agency that workers express towards platforms. They are gig work’s capacity to shape worker agency and the role of labor market positionality, which limits worker agency potential. According to their study among Australian food-delivery workers, the agency is shaped by workers competing against each other to maximize their income, for example, by working simultaneously for multiple platforms (Barratt et al., 2020). On the other hand, the position that these workers are in due to poor labor market alternatives (since most of them are migrants), in addition to the low entry barriers, lowers their labor market expectations (Barratt et al., 2020). Çiğdem (2022) recognizes unemployment as a push factor towards self-employment but notes that the majority of workers enter the gig economy voluntarily. Dunn (2020), however, mentions several categories of gig workers’ motivations: some work out of pure necessity, others to embrace the freelance lifestyle and some others to earn extra income. Kervola et al. (2022) found that there is no shortage in the workforce among crowdshipping companies that promote the freedom of workers, while last-mile and logistics service providers suffer from labor shortages. Platform companies such as Uber have promoted themselves as socially responsible actors providing opportunities for micro-entrepreneurship (Ahsan, 2020).

However, Wood and Lehdonvirta (2021) question the view that labor platforms would be neutral marketplaces because the companies control demand and coordinate important functions, information and contracting costs. Thus, workers are subordinated towards platforms even though they experience enhanced agency towards clients, which Wood and Lehdonvirta (2021) call “subordinated agency” driving workers to support self-employed status but also to pursue collective action. Maffie (2023) agrees and shows how platform companies take a layered approach in actively shaping market forces and pressuring workers to prefer independent contractor status through the use of “visible hands,” which are information control, uneven job distribution and temporal insulation from market risk. Castillo-Villar et al. (2024) conclude that algorithmic control platforms use, and workers being forced to be entrepreneurs, are the two exploitive tactics that ridesharing companies use.

In exchange for freedom, self-employed workers are responsible for themselves, and companies are accused by many scholars of leaving workers without social protection (Fleming, 2017; van Doorn, 2017) and making them work under poor working conditions (Moncef and Monnet Dupuy, 2021). This is the reason why an increasing number of workers would also prefer employment over entrepreneurship (Regan and Christie, 2023). Both Ahsan (2020) and Çiğdem (2022) argue that, in reality, freedom and flexibility are only a myth or an illusion, since there is only a little freedom for the workers. Sun et al. (2021) have reported the de-flexibilization of Chinese food delivery work, which means that flexibility is, in fact, decreasing and workers are pushed into working full-time with fixed hours. As a result, these workers are victims of “sticky labor,” which narrows down their opportunities in the job market and confines and disciplines workers (Sun et al., 2021).

Popan (2021) uses the term “flexploitation” to describe the exploitation that platforms exercise when they advertise to offer freedom to workers in a precarious situation when simultaneously there is a high rate of uncertainty related to income and work schedule. As Kuhn and Maleki (2017) note, if the firm has control over pay, evaluates performance and rewards and punishes workers, worker autonomy is low. Vieira (2021) concludes that, even though couriers are not free or autonomous, they cherish the self-employment status in opposition to what the broader labor market offers them—temporary work with a low wage and inflexibility. There is, however, variation in worker autonomy between platforms. For example, some platforms allow workers to go online whenever they want, while others require preliminary shift booking that gives priority to workers with a higher rating.

As a response to the ongoing debate on the employment status of workers, there has risen the “dependent contractor” category, which offers food delivery workers some limited access to social and employment protections (Kuhn and Maleki, 2017; Rolf et al., 2022). Examples of such practices include a minimum hourly wage guarantee and holiday pay (Rolf et al., 2022). In a similar vein, Lee (2022) reports of a “quasi-employee” status in Taiwan, where a hybrid logic of employment relations is applied to food delivery couriers working neither as employees nor as self-employed. In Finland, food delivery couriers have started to organize and have become part of a trade union (Palvelualojen ammattiliitto PAM, 2023), which has traditionally been possible for employees only. van Doorn et al. (2022), however, point out that a shift from self-employment to employment does not offer a solution to low-wage workers if their livelihoods and dignity are not secured. Dubal (2019) states that the whole question of self-employment or employment in a platform economy is fundamentally wrong because the focus of the discussion should be on what protections workers need. This seems to be the way that some countries are already proceeding, even though the discussion over employment and self-employment remains on the surface.

To summarize the discussion about the employment status of couriers, we compare the recent literature to the research questions of our study. No hypothesis can be made of research question 1, because to our best knowledge, such research does not exist that would elaborate on the couriers’ preference of employment status and there are only a handful of studies that discuss gig workers’ employment status more closely. Most of those studies concentrate on couriers’ motivations towards self-employment. Work-related factors such as flexibility and freedom in relation to time and income, and the economic empowerment or self-development rhetoric seem to explain best this preference. It could be hypothesized in relation to research questions 2 and 3 that the explanatory factors behind couriers’ preference of self-employment would include flexibility and freedom in relation to the use of time. The literature gives also other explanations to gig workers preferring self-employment such as competition between workers, poor labor market opportunities, low entry barriers, unemployment, and control and subordination exercised by platforms. Thus, the literature presents a rather critical view of the employment status in relation to gig work.

## 3 Data and methods

This study examined which employment status food delivery couriers prefer and what factors explain their willingness to work either as self-employed or as employees. The analysis is based on a survey commissioned by Wolt and conducted by an independent market research company Taloustutkimus for Wolt couriers from 7–15 April 2021. Taloustutkimus has been conducting research since 1971 and is the second largest full-service market research company in Finland. The company is part of the World Independent Network of Market Research. Wolt currently operates in 25 countries and has around 200,000 couriers worldwide (Raeste, 2023). Wolt was established in 2014, and in 2022, they joined forces with US-based DoorDash (Wolt, 2023).

As an independent research institute, Taloustutkimus was responsible for the design of the questionnaire, its neutrality and the instructions given to the respondents. The survey form and instructions can be found in Appendix 1. Taloustutkimus gathered the data through an e-mail survey that included both closed-ended and open-ended questions. The survey was sent to 3,674 Wolt couriers in Finland, of whom 1,539 (42%) responded. Invitations to the survey were sent from the Taloustutkimus address and the responses were routed back to Taloustutkimus, without Wolt having access to the survey at any point. The survey answer links were generated uniquely so that one person could answer only once. The survey could be answered in either Finnish or English. The survey was anonymous.

The authors of this article received access to the original and unedited survey data from Taloustutkimus and permission from Wolt for its scientific use. The analysis of the survey was carried out using IBM SPSS software. The first research question was used to determine which employment status food delivery couriers preferred. The answer was sought through the question (Q7): “Would you rather work as a Wolt courier as a contractor (i.e., self-employed) or as an employee?” When the respondents had answered the question for the first time (Q7), changing the given answer was prevented. They were then given additional information about being self-employed and being an employee (Appendix 1). After the additional information, the same question was asked again (Q8) to see how the additional information provided changed their preferences?

The second research question investigated which factors could explain the couriers’ willingness to work as self-employed or as an employee. This was investigated by using a logistic regression (LR) analysis and cross-tabulation. LR was selected as the analysis method because the dependent variable was binary (Mood, 2010; Stoltzfus, 2011). In this study, the LR model was used to better understand which variables affected the couriers’ willingness to work either as self-employed or as employees and how strong the effect of each variable was. In the LR, question Q7 was defined as the dependent variable. Only answers in which the respondent wanted to work either as self-employed or as an employee were included in the data. Answers in which the respondent indicated they were indifferent or uncertain were excluded. Those answers stating that the respondent had not made any Wolt deliveries were also excluded. The independent variables used in the original model are shown in Table 1.

**Table 1:** The independent variables used in the logistic regression analysis.

Categorical variables	*n*	% share				
Age (Q14)						
16–34	609	61.5				
35 or more	382	38.5				
Total	991					
Education (Q15)						
Lower than university degree	349	35.2				
University degree	642	64.8				
Total	991					
Working time as a Wolt courier (Q17)						
0–6 months	409	41.3				
7 months or more	582	58.7				
Total	991					
Share of Wolt courier income of total income (Q5)						
0–50% and I’m not sure	514	51.9				
51–100%	477	48.1				
Total	991					
Own choice to work as a Wolt courier (Q6)						
I work as a Wolt courier because I cannot get any other work	186	18.8				
It’s my own choice to work as a Wolt courier	805	81.2				
Total	991					
Weekly working time (Q18)						
0–30 h	493	49.7				
31 h or more	498	50.3				
Total	991					
Working part-time or full-time (Q19)						
I have done deliveries part-time	467	47.1				
I have done deliveries full-time	524	52.9				
Total	991					

Continuous variables	*n*	Mean	Std. dev	Min	Max	Type
Satisfaction with income as Wolt’s courier (Q2)	991	3.82	1.03	1	5	Likert
Job satisfaction in general as Wolt’s courier (Q3)	991	4.17	0.91	1	5	Likert
Freedom to choose own workload, time and delivery vehicle (Q10.2)	991	2.91	1.09	1	4	Likert
Valuing freedom over job stability (Q10.4)	991	3.22	1.00	1	4	Likert
Freedom to adjust own income (Q11.1)	991	3.69	0.67	1	4	Likert
Right to refuse delivery tasks offered (Q11.6)	991	3.05	1.13	1	4	Likert

LR analysis was possible because the Q2, Q3, Q10.2, Q10.4, Q11.1 and Q11.6 variables were continuous using Likert scales, and the categorical variables Q14, Q15, Q17, Q5, Q18 and Q19 were converted to binary variables. Q10.2, Q10.4, Q11.1 and Q11.6 included the option “I cannot say” in the original survey. These responses were removed from the analysis so as not to bias the results. Collinearity tolerance was used for categorical variables and VIF for continuous variables to measure how much beta coefficients were affected by the presence of other independent variables in the model. In other words, they measure the compatibility between variables (Midi et al., 2010). Based on those indicators, there were no compatibility problems.

The model was created using the backward stepwise LR method, where the original model contains all possible variables. The variable with the least statistical significance is subtracted from the model one at a time (Stoltzfus, 2011). The Hosmer and Lemeshow test was used to test the validity of the model (Stoltzfus, 2011), and the Nagelkerke R-Square was used to describe the proportion of the total variance of the dependent variable that can be explained by the independent variables in the model (Mondal and Mandal, 2018). The results obtained using backward stepwise LR were also examined using cross-tabulation to increase the understanding. For cross-tabulation, Pearson’s Chi Square was used as a test of statistical significance (Greenland, 2019).

The third research question sought an answer to the question of what couriers value the most as self-employed persons and as employees. The method used was a content analysis of the open-ended questions in the survey. The questions were: “Why would you prefer to work as a contractor (i.e., self-employed)?” (Q7A) and “Why would you prefer to work as an employee?” (Q7B). 789 verbal answers were given to the question Q7A and 270 verbal answers to the question Q7B. The data was coded, and an observation table was built to analyze the data. One answer could result in several observations. Categories and subcategories were formed from the observations.

## 4 Results

To understand the results, it is important to perceive the background factors of the respondents. 52.9% of the respondents worked full-time and 47.1% part-time. 64.8% had a university degree and 61.5% were 16–34 years old. 58.7% had worked for more than 6 months. About half of the respondents carried out Wolt deliveries for more than 30 h a week, and for 48.1% of them, the Wolt courier income was more than half of their total income. 81.2% worked as a courier because they wanted to and 18.8% because they had not found other jobs. It needs to be pointed out that the survey of Taloustutkimus did not ask about the nationality, gender, family background or occupational background of the respondents, so these could not be included in the explanatory variables. However, courier work in Finland relies heavily on labor from foreign backgrounds (Pärnänen, 2023). A more detailed profile of respondents and the values of the continuous variables can be found in Table 1.

### 4.1 Couriers’ preferred employment status

**Figure 1:**
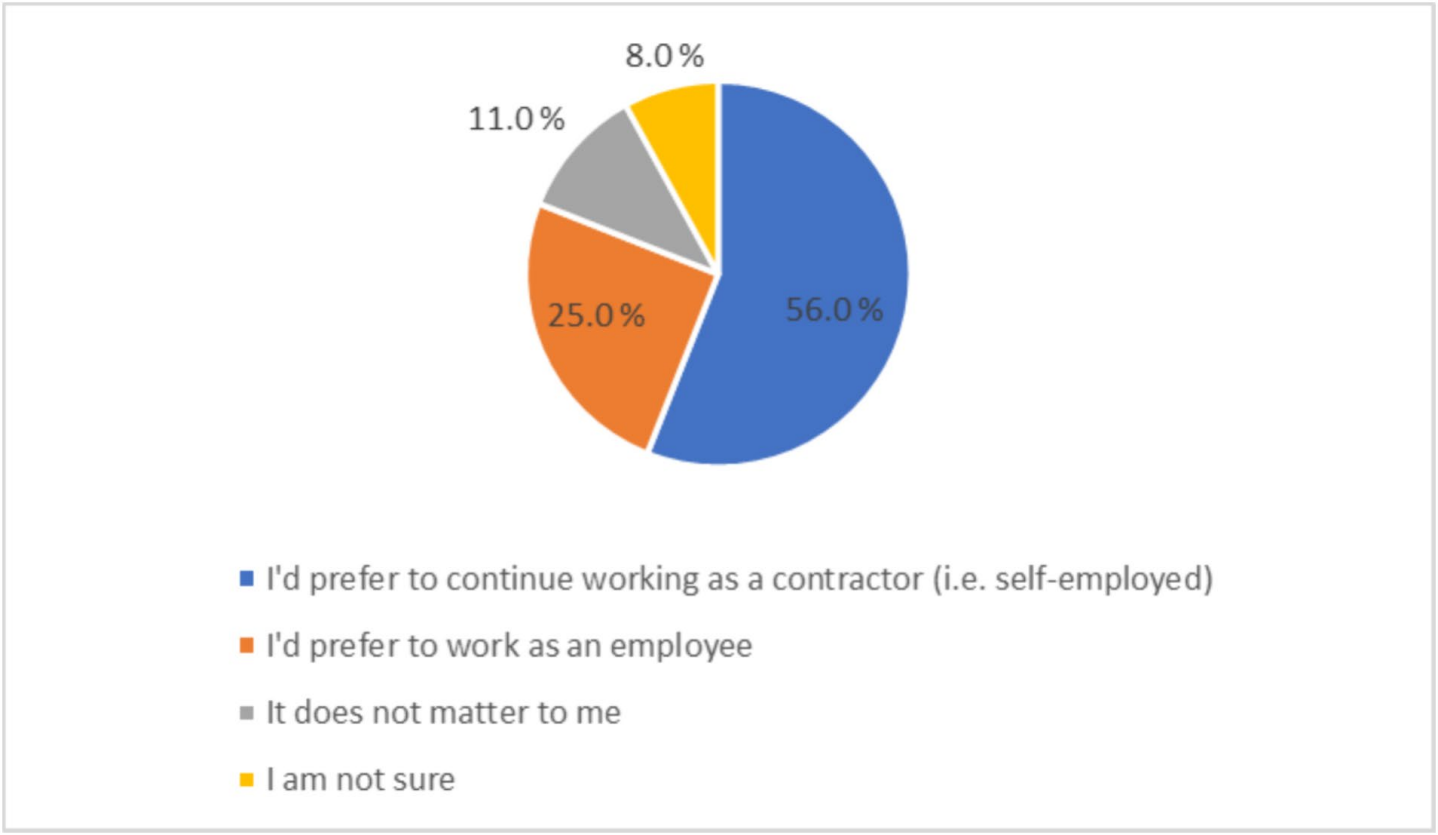
Couriers’ preferred employment status (*n* = 1,539).

56.0% of Wolt couriers wanted to be self-employed, while 25.0% would prefer to work as employees, 11.0% did not care and 8.0% were not sure (Figure 1). Thus, of the 1,539 respondents, 1,246 had an opinion about being either self-employed or an employee: 69.2% indicated they would prefer to be self-employed, and 30.8% would prefer to be in an employment relationship.

**Figure 2:**
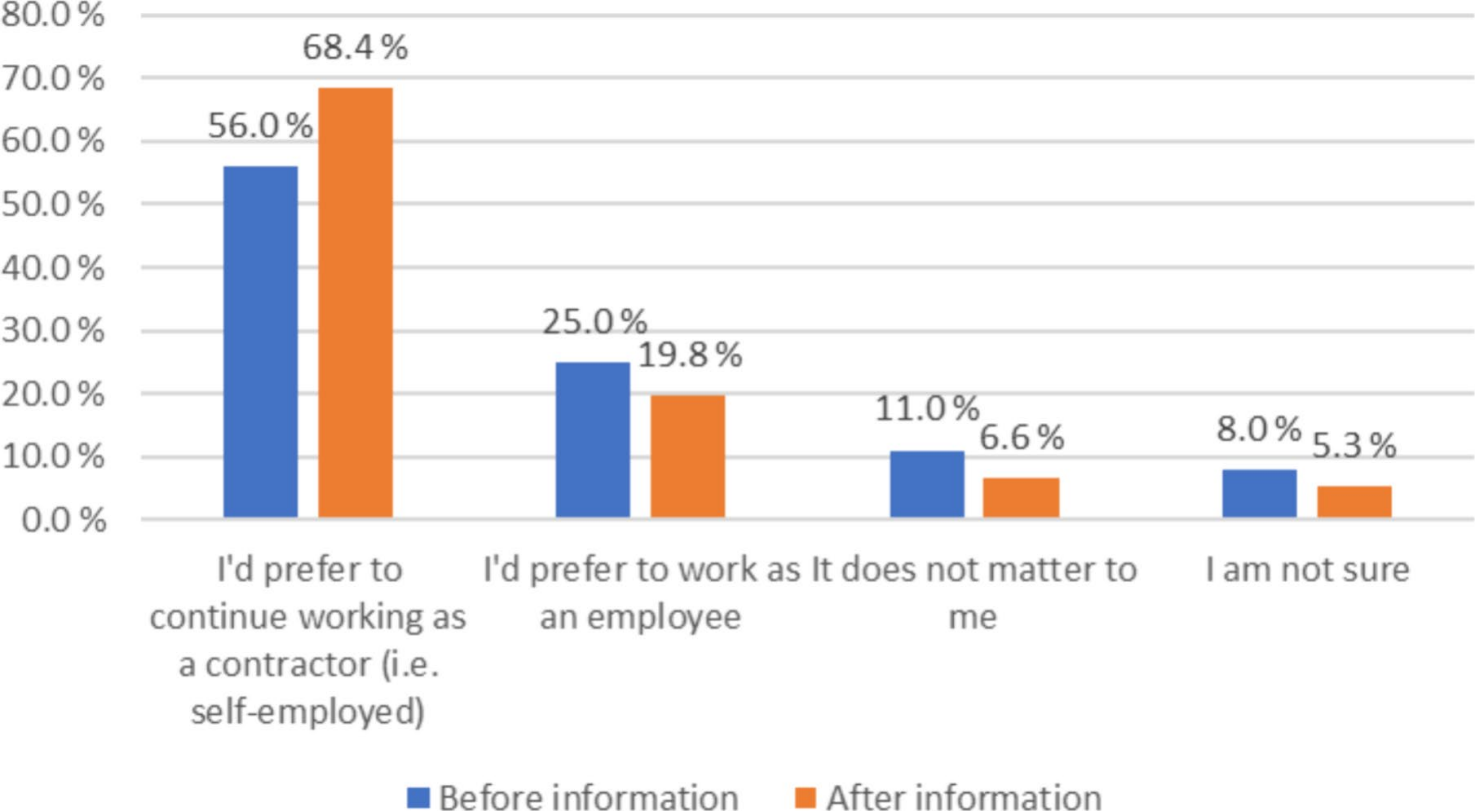
The effect of the additional information on responses (*n* = 1,539).

After the respondents had answered the question asking whether they would prefer to be self-employed or employed, they were given additional information with examples of both self-employment and working as an employee. This information described the effects of the choice on their gross income, employee benefits, working hours, having a supervisor, choosing, and refusing jobs, choosing a vehicle, setting work goals, and doing other work. For more information, see Appendix 1. The additional information increased the number of those who wanted to continue as self-employed from 56.0 to 68.4% (Figure 2). The number of people who wanted to work as an employee decreased from 25.0 to 19.8%. The number of indifferent and uncertain responses also decreased significantly.

### 4.2 Work related factors explaining the willingness to work as self-employed or as an employee

Based on the backward stepwise LR analysis, seven variables were included in the final model as shown in Table 2. The value of the Hosmel and Lemeshow test was 0.82, and the Nagelkerke value was 0.31. In the cross-tabulation, Pearson’s chi-square was <0.001 for most and < 0.05 for all, as shown in Table 3.

**Table 2:** Logistic regression model related to the factors explaining willingness to work as self-employed or as an employee (*n* = 991); Hosmer and Lemeshow 0.82; Nagelkerke 0.31.

Factor	B	S.E.	Wald	df	Sig.	Exp(B)
Valuing freedom over job stability	0.905	0.085	112.705	1	<0.001	2.473
Working time as a Wolt courier	0.770	0.164	22.016	1	<0.001	2.159
Age	0.465	0.170	7.512	1	0.006	1.592
Own choice to work as a Wolt courier	0.488	0.200	5.939	1	0.015	1.629
Right to refuse delivery tasks offered	0.146	0.073	4.038	1	0.044	1.158
Share of Wolt courier income of total income	0.315	0.165	3.652	1	0.056	1.370
Freedom to choose own workload, time and delivery vehicle	0.134	0.078	2.958	1	0.085	1.144
Constant	−3.891	0.391	98.967	1	<0.01	0.020

**Table 3:** Cross-tabulation of factors explaining willingness to work as self-employed or as an employee.

Factor	Classes	Willingness self-employed (%)	Willingness employee (%)	Total (%)	*n*
Valuing freedom over stability (Pearson X^2^ < 0.001)	1. I disagree	27.7	72.3	100.0	119
	2. I somewhat disagree	37.2	62.8	100.0	129
	3. I somewhat agree	72.8	27.2	100.0	309
	4. I agree	83.5	16.5	100.0	623
	Total	70.0	30.0	100.0	1,180
Working time as a Wolt courier (Pearson X^2^ < 0.001)	0–3 months	57.8	42.2	100.0	296
	4–6 months	63.4	36.6	100.0	224
	7–12 months	73.8	26.3	100.0	240
	12–24 months	75.6	24.4	100.0	242
	25 months or more	77.5	22.5	100.0	244
	Total	69.2	30.8	100.0	1,246
Own choice to work as a Wolt courier (Pearson X^2^ < 0.001)	I work as a Wolt courier because I cannot get other jobs	57.0	43.0	100.0	223
	I work as a Wolt courier because I have chosen to do so	71.8	28.2	100.0	1,023
	Total	69.2	30.8	100.0	1,246
Right to refuse an offered gig (Pearson X^2^ < 0.001)	1. I disagree	54.9	45.1	100.0	206
	2. I somewhat disagree	67.2	32.8	100.0	131
	3. I somewhat agree	72.0	28.0	100.0	282
	4. I agree	74.6	25.4	100.0	552
	Total	69.7	30.3	100.0	1,171
Freedom to choose own workload, time and delivery vehicle (Pearson X^2^ < 0.001)	1. I disagree	45.2	54.8	100.0	31
	2. I somewhat disagree	47.9	52.1	100.0	48
	3. I somewhat agree	67.6	32.4	100.0	182
	4. I agree	71.6	28.4	100.0	962
	Total	69.4	30.6	100.0	1,223
Age (Pearson X^2^ = 0.008)	16–24	65.2	34.8	100.0	155
	25–34	65.7	34.3	100.0	604
	35–44	75.1	24.9	100.0	398
	45+	73.0	27.0	100.0	89
	Total	69.2	30.8	100.0	1,246
Share of Wolt courier income of total income (Pearson X^2^ = 0.011)	I cannot say	62.9	37.1	100.0	197
	0–25%	64.6	35.4	100.0	226
	26–50%	67.4	32.6	100.0	242
	51–75%	71.5	28.5	100.0	214
	76–100%	75.2	24.8	100.0	367
	Total	69.2	30.8	100.0	1,246

Valuing freedom over job stability was the strongest explanatory factor, with an odds ratio of 2.47. 83.5% of those who agreed with the statement wanted to be self-employed, and 72.3% of those who disagreed wanted to be employees. Of those respondents for whom it was important to have the right to refuse the tasks offered, 74.6% wanted to be self-employed. The odds ratio was 1.16. The same was seen for the statement about the freedom to choose the workload, time, and delivery vehicle. 71.6% of those for whom freedom was important wanted to be self-employed, and 54.6% of those for whom it was not important wanted to be self-employed. The odds ratio was 1.14.

In terms of the duration of the courier work, the willingness to be self-employed was stronger the longer one had worked as a Wolt courier. The odds ratio was 2.16. The highest willingness was among those who had worked for more than 25 months, and the lowest was among those who had worked for 0–3 months. Of those who had chosen courier work, the willingness to be self-employed was 71.8%. Even among those who worked as couriers because they had not found other jobs, the desire to be self-employed was greater than the desire to be an employee. The results also show that the willingness to be self-employed increased with age, with an odds ratio of 1.59. It was strongest in the 35–44 age group. For people over 45, it was a little lower than in the 35–44 age group but still at a high level. Regarding earnings, those who received all or almost all their income from working as a Wolt courier had the greatest desire to be self-employed.

### 4.3 The most valued things as self-employed

**Figure 3:**
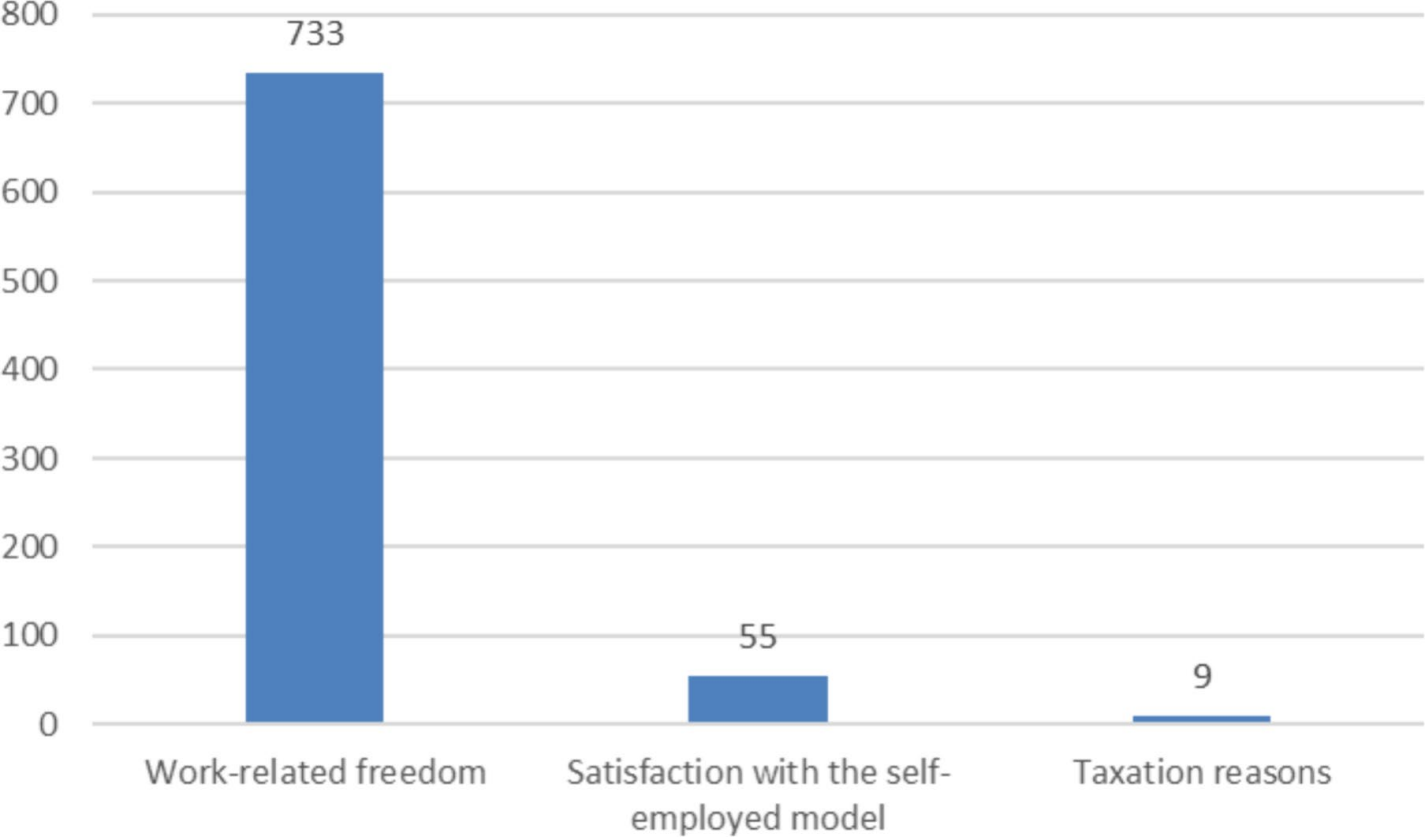
The most valued things about working as self-employed—the number of verbal comments.

Based on the verbal answers, three categories were formed that explain why couriers preferred being self-employed rather than working as employees. The categories were work-related freedom, satisfaction with the self-employment model and taxation reasons (Figure 3). Among the categories, work-related freedom was clearly the most significant factor, with 733 comments. Satisfaction with the self-employed model received 55 comments and taxation reasons got 9 comments. The categories are not mutually exclusive. For example, in the case of freedom and satisfaction, the former is more of a cause and the latter a consequence.

**Figure 4:**
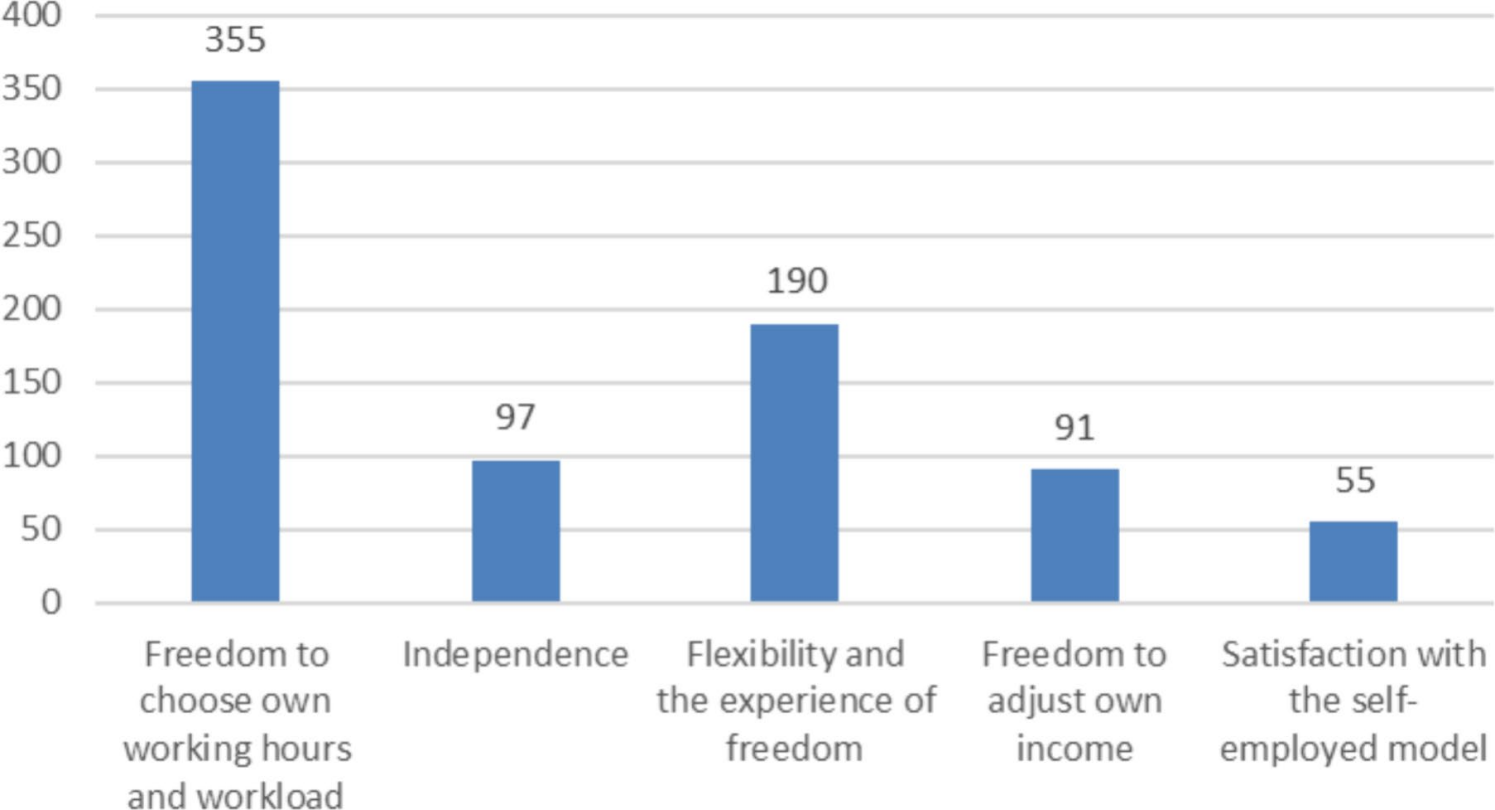
Verbal comments relating to work-related freedom.

Since work-related freedom was clearly the most valued thing, we looked more closely at how the respondents expressed work-related freedom. Verbal comments brought up the freedom to choose one’s own working hours and workload, flexibility and the experience of freedom, independence, and the freedom to adjust one’s income (Figure 4). Of these, the freedom to choose the working hours and workload received the most mentions, appearing in 355 responses.

*“The reason why I came to work for Wolt is freedom. I can come to work at any time during opening hours and leave at any time” (Respondent 565).*

Flexibility and the experience of freedom were mentioned the second most. The experience of freedom was commented on without defining it more precisely. Some said that if courier work was performed as an employee, it would no longer be interesting.

*“Flexibility and freedom suit me better. Working as a courier would not interest me in the form of an employment relationship” (Respondent 834).*

In connection with independence, the desire to be one’s own boss was emphasized. Many respondents also highlighted their desire to be an entrepreneur. In connection to independence, the possibility to refuse a job was mentioned.

*“I am my own boss. I can choose my schedule. I can also choose whether to pick or decline a task. I like the freedom that comes with it” (Respondent 1513).*

In connection with freedom, the freedom to adjust one’s own income level as needed was brought up. Benefiting from one’s own diligence and hard work also came up.

*“I am in control of my time and net income per month” (Respondent 1,125).*

*“I can see the benefit of my hard work” (Respondent 37).*

### 4.4 The most valued things as an employee

**Figure 5:**
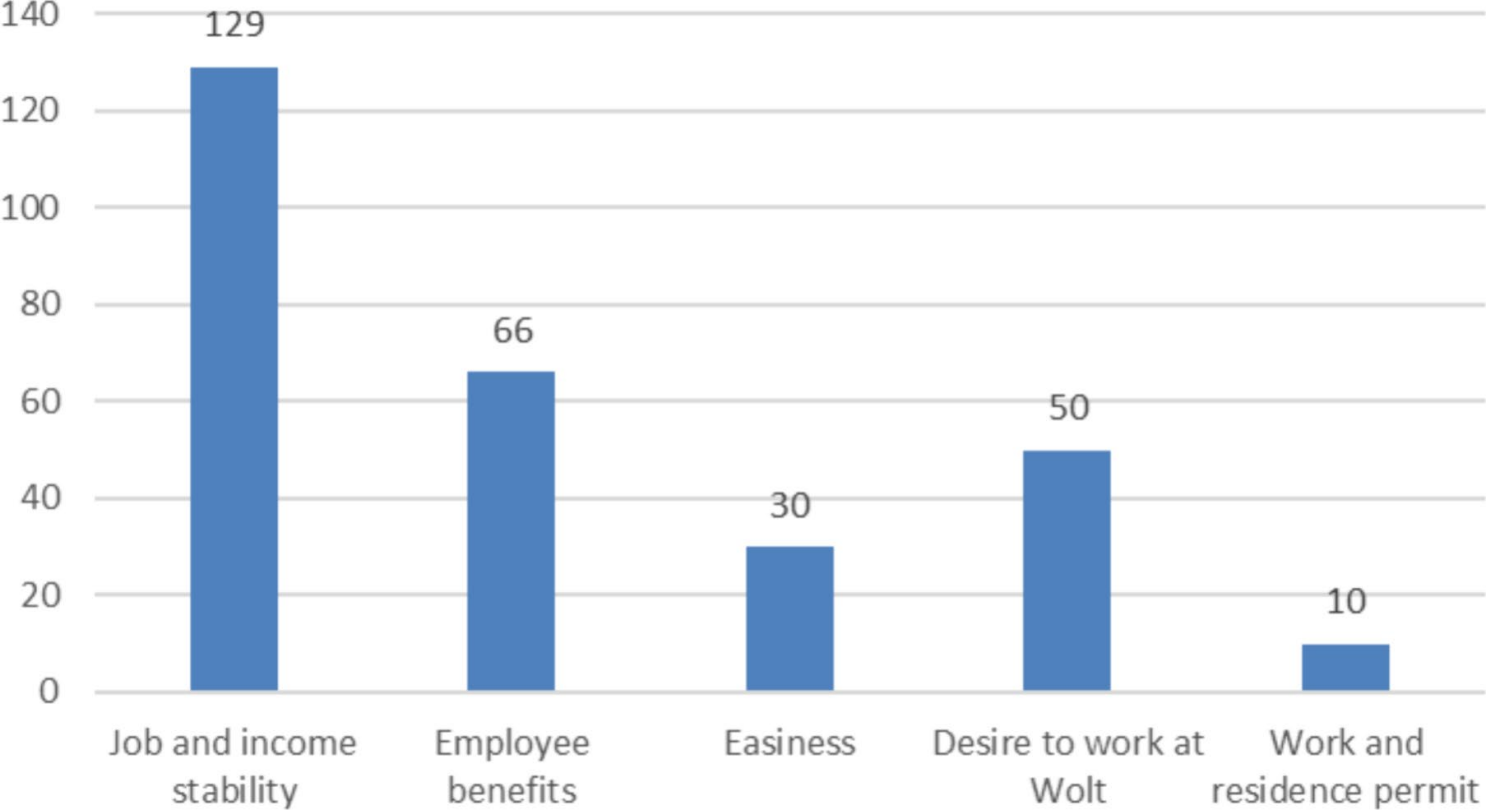
The most valued things as an employee—the number of verbal comments.

Based on the written responses, five categories emerged as to why couriers preferred to work as employees. The categories were job and income stability, employee benefits, ease, the desire to work at Wolt, and work and residence permits (Figure 5). Job and income stability was the most important category with 129 mentions. It emphasized the importance of regular work and income to secure a future. For a self-employed person, the fluctuation in the number of gigs caused uncertainty. Some respondents commented that Wolt has increased the number of couriers, which has led to longer waiting times for gigs and thus to lower earnings.

*“You will be sure about your income so you will not be on pressure” (Respondent 1,009).*

*“Recently, Wolt decided to over-employ more than the optimal number of couriers. As such, couriers now struggle to earn enough money to get by. As an employee, I would be sure how much I would earn at the end of each month. If Wolt ensures that the city is not flooded with an over-optimal number of couriers, I would prefer a contractor (self-employed) relationship” (Respondent 440).*

The second most important factor was employee benefits with 66 responses. Respondents mentioned financial benefits such as sick pay and holiday pay. In addition, health-related issues such as accident insurance and occupational health care were also highlighted. Annual leave and pension rights were also raised.

*“While working, the employee would receive the benefits due to the employee, such as occupational health care, annual leave, sick pay” (Respondent 1,529).*

Satisfaction with Wolt and the company’s practices was one of the reasons why some of the respondents would have wanted to work for Wolt as an employee. In terms of ease, the desire to focus on performing work rather than on entrepreneurial issues such as paperwork, taxes and bureaucracy was highlighted.

*“Because I have always loved to work with Wolt” (Respondent 457).*

*“No need to take on extra pain. Work and pay, that’s all” (Respondent 429).*

Here we have presented the results of the study. The results will be discussed in the following section together with the previous literature.

## 5 Discussion

The aim of this article was to examine couriers’ preferred employment status and factors explaining their opinions. The first research question elaborated on which employment status food delivery couriers preferred. According to our research, 56.0% of Wolt couriers wanted to be self-employed, and 25.0% wanted to work as employees. When “It does not matter to me” and “I am not sure” answers were removed, the distribution was 69.2% for self-employment and 30.8% for employment. This result is in line with the situation in Spain, where platform workers protested against the Spanish government that wanted to make them employees (Vieira, 2021). In the Spanish context, however, no research was conducted on how the opinions were divided between self-employment and employment and the reason behind supporting self-employment laid in the broader employment situation in the society. Regan and Christie (2023) claim that food delivery couriers increasingly insist on being treated as employees. This does not seem to be the case for Wolt couriers in Finland.

However, knowing that most of the Wolt couriers are from immigrant backgrounds, the question can be asked, to what extent they can take a stand on the different employment options in the Finnish society. Finland is a strong welfare state with good social support in case of unemployment. According to the employment survey of Statistics Finland in 2022 (Sutela, 2023), it is more common for immigrants in Finland to have atypical employment relations such as temporary agency work, part time work or to be light entrepreneurs compared to the Finnish population. Compared to the EU, the immigrant employment rate is above average in Finland (Sutela, 2023). However, there is no clear-cut explanation from the societal background factors as to why couriers in our research prefer self-employment.

The first research question also examined how the additional information influenced the couriers’ responses. It clarified the difference between self-employment and employment by providing examples of how each choice impacted the working conditions of the couriers. After receiving additional information, 68.4% of couriers wanted to be self-employed, and 19.8% would have preferred to be employed, which increased the willingness to work as self-employed by 12.4 percentage points. In the additional information provided, it would have been good to mention that the employment relationship guarantees a minimum wage. The additional information also states that the employment relationship has a lower gross income per delivery and per average hour. While this may be true in principle, if there are not enough delivery tasks, the reverse may also be true. Due to these inaccuracies, we emphasize the results of the survey, which were obtained before respondents were given additional information.

Our second research question examined the factors explaining the couriers’ willingness to be self-employed or to work as an employee. Valuing freedom and flexibility was the most significant explanatory factor in our research. This result is also in line with earlier studies (Ivanova et al., 2018; Mäntymäki et al., 2019; Wood et al., 2019; Vieira, 2021; Çiğdem, 2022; Kervola et al., 2022). In our study, those for whom freedom and flexibility were important wanted to be self-employed, and those for whom job stability was important wanted to be employed. The desire for freedom and flexibility was accompanied by the right to refuse gigs offered and the freedom to choose the amount of work, the working hours, and the delivery vehicles. We hypothesized that freedom and flexibility in relation to the use of time would be the explanatory factor behind self-employment and our research confirms this hypothesis. In a survey of 6,811 Wolt couriers from 14 European countries, 90% said it was important or somewhat important that they could decide which tasks to accept and reject, and 93% considered important or somewhat important that they could choose when to make deliveries (Taloustutkimus, 2023). According to Wolt’s CEO Kuusi, couriers reject 50–60% of delivery tasks offered (Raeste, 2023), which also shows that this right is important to couriers.

Wolt seems to have chosen a softer strategy for platform work: couriers can reach to customer support personnel who help them with problems while delivering, the compensation system is transparent, and they can even refuse gigs offered to them without sanctions (Kusk and Bossen, 2022). Even though Çiğdem (2022) claims that freedom and flexibility are only a myth in case of platform workers, Wolt couriers seem to have been given more freedom than workers working for other platform companies. They do not have to book shifts in advance and can start working whenever they like and the monitoring does not seem to bother them (Kusk and Bossen, 2022). It is also probable that Wolt has done its part in convincing couriers about the benefits of self-employment and thus emphasize freedom as the key distinction compared to employment.

The duration of courier work was also an important explanatory factor. The longer couriers had been working for Wolt, the greater was their desire to be self-employed. The correlation between the two is likely to be strengthened by the fact that some of those who value employment have stopped working because the working model did not meet their expectations. A personal choice was also a significant explanatory factor. If courier work was a personal choice, self-employment was popular, which is in line with Gregory’s (2021) notion about the entrepreneurship discussion promoting the opportunity to determine one’s own life. It seems that Wolt couriers do not see themselves as victims that are exploited, which the previous research brings out as a concern (Popan, 2021; Sun et al., 2021).

In terms of income, the greater the proportion of their total income was from Wolt, the more willing they were to be self-employed. The result is surprising because, intuitively, one would imagine that those who earn all or almost all their income working as a Wolt courier would want to be employees because in gig work, it may be hard to support oneself. This result also goes against the argument about platform dependency proposed by Schor et al. (2020). It is likely that these respondents have learned to sustain themselves as couriers, they are satisfied with their situation, and they see no need to change their employment status. They experience “individual economic empowerment” and therefore want to be self-employed (Martin, 2016). Temporal factors that Kusk and Bossen (2022) consider, can explain the connection between income and self-employment. The study was performed during COVID–19 pandemic, which caused an economic boom in the food delivery sector as people ordered food to their homes instead of going to the restaurants to eat. It was easier for the couriers to earn their living than before. Thus, there are several factors explaining food delivery couriers’ willingness to be self-employed brought forward by our research that either agree or contradict with previous research.

Our third research question sought to understand what couriers valued most in being self-employed or employed. The verbal results were consistent with the statistical data. Those who wanted to be self-employed valued the freedom of choice concerning when and how much to work, the flexibility and the experience of freedom and independence. As noted above, Wolt couriers appreciate freedom that seems to be a genuine feeling for them, which explains their desire to be self-employed. Wolt’s soft policy towards couriers contributes to this experience, as noted earlier. Also, the response to this research question is in line with our hypothesis about couriers valuing freedom and flexibility in relation to the use of time.

Those Wolt couriers who wanted to work as employees valued job and income stability the most. The most uncertainty was caused by the fluctuation in the number of gigs and its impact on earnings, which was in line with the findings of earlier studies (Duggan et al., 2021; Popan, 2021). In a downturn, when the number of delivery tasks is reduced, there is a risk that the number of couriers will be too high compared to demand. As a result, couriers are forced to spend more time waiting for gigs, leading to a drop in earnings. According to Wolt’s CEO Kuusi, there have been challenges in balancing the right number of couriers (Raeste, 2023). He also says it has been a value issue for Wolt that courier contracts have not been cancelled due to reduced demand. The flip side of this is that when demand for food delivery services falls, so do the earnings of couriers. This naturally creates a desire for a steady income. However, it is worth noting that a change in employment status does not eliminate problems caused by economic cycles and fluctuations in demand. In an employment relationship during a downturn, an individual courier could face a lay-off or dismissal. In addition to job stability, employee benefits such as sick pay, holiday pay, occupational health care, annual leave and pension accrual were considered important. The result is consistent with the results of Fleming (2017), van Doorn (2017) and Moncef and Monnet Dupuy (2021).

Thus, the situation of Wolt couriers appears to be in line with explanations previous studies gave to couriers’ willingness to work as self-employed, which is the desire for flexibility and freedom. However, most of the previous studies present a presupposition that couriers would prefer employment. In this research, couriers seem to have adopted the identity of an entrepreneur and thus feel free to make their own decisions regarding their work.

## 6 Conclusion

### 6.1 Summary and implications

This research discusses food delivery couriers’ preferred employment status, and which factors explain their opinions. The analysis is based on a survey of Wolt couriers in Finland. For science, this article provides new valuable information about the food delivery couriers’ preferred employment status. The results show that a clear majority of the couriers who participated in this study, preferred self-employment, which is significant for industrial relations. The strongest explanation for this opinion was the appreciation of freedom and flexibility at work. Those who valued security over freedom, wanted to be employees. Freedom was associated with the right to refuse delivery tasks offered and the freedom to choose the amount of work, the working hours, and the delivery vehicle. The willingness for self-employment was also increased by the duration of courier work, the courier’s own choice to work as a courier, and age. Those who wanted to work as employees emphasized job and income stability and employee benefits such as sick pay, holiday pay and annual leave.

An implication of this article is the realization that the self-employment model seems to work well when there are enough delivery tasks available for couriers. This poses a challenge for companies to balance the number of couriers when demand falls or is lower than expected. Balancing should be done in the most socially responsible way possible. If balancing is left to the labor market, it will create dissatisfaction both with the couriers and society. The research also shows that it seems possible to organize gig work in a way that allows for genuine freedom and flexibility. By enabling freedom, the workforce is available even if the industry would otherwise suffer from labor shortages.

A political implication of the article is that when applying EU regulations or forming legislation regarding platform work, they should not neglect the desire of platform workers for freedom and flexibility in their work. Thus, the regulation should consider the various situations that platform workers have, others preferring security and others flexibility. The legislation should take both needs into account.

### 6.2 Limitations and further research

The study was based on a survey conducted by an independent research institute and the authors of the article had access to the unedited original survey data. The authors of the article carefully evaluated the neutrality of the questions in the survey and the associated instructions. Even though Wolt commissioned the survey, it does not change the results, even though it would have been in their interest also to publish them. This study was limited by its focus on only one company, and the results do not give a full picture of the entire industry. On the other hand, the study gives a comprehensive picture of Wolt in Finland because the response rate was high. Another limitation is that the questionnaire did not ask about gender, nationality, family background or work background. Another limitation is that the study was conducted during a boom in the food delivery business.

As a subject for further research, it would be interesting to repeat the same study for Wolt couriers in different economic situations and compare the results. It would also be good to conduct a similar study for other food delivery companies to compare how their practices and different ways of using algorithmic management affect the results. A comparative perspective between countries would also be interesting. Further research is also needed on drivers’ experiences of fairness in platform-based transport companies and societal background factors influencing the immigrant workers’ choices in the labor market in Finland.

## Data availability statement

The data analyzed in this study is subject to the following licenses/restrictions: the study is based on a survey carried out by an independent research institute (Taloustutkimus) for a company (Wolt). The authors of this article received access to the original and unedited survey data from Taloustutkimus and permission from Wolt for its scientific use. However, the researchers do not have permission to publish the original raw data. Requests to access these datasets should be directed to juhani.mykkanen@wolt.com.

## Ethics statement

Ethical approval was not required for the studies involving humans because it was not applicable. The survey did not deviate from the principle of informed consent, nor contain any of the criteria for ethical review by the Finnish National Board on Research Integrity. The studies were conducted in accordance with the local legislation and institutional requirements. The participants provided their written informed consent to participate in this study.

## Author contributions

HK: Conceptualization, Data curation, Formal analysis, Funding acquisition, Investigation, Methodology, Project administration, Resources, Software, Validation, Visualization, Writing – original draft, Writing – review & editing. SH: Writing – original draft, Writing – review & editing. EK: Writing – original draft, Writing – review & editing. HL: Conceptualization, Methodology, Supervision, Writing – original draft, Writing – review & editing.
