# Supplementary material for: Flexibility and freedom suit me better: food delivery couriers’ preferred employment status
Source: Front Sociol. 2024 Jun 17;9:1415852. doi: 10.3389/fsoc.2024.1415852 (PMC11229694; doi:10.3389/fsoc.2024.1415852)
Supplement: Supplementary file 1 [file Data_Sheet_1.PDF]

Appendix 1: Survey form

For background

The questions below aim to find out what Wolt's courier partners ("Wolt couriers") think about partnering with Wolt. All results will be processed anonymously. Wolt or anyone else will not be able to tell who has answered the survey, or what a specific person has answered.

Satisfaction and working situation

How satisfied are you with your partnership with Wolt? (Q1)
5 = very satisfied – 1 = very unsatisfied

How satisfied are you with your income as a Wolt courier? (Q2)
5 = very satisfied – 1 = very unsatisfied

How satisfied are you in general with working as a Wolt courier? (Q3)
5 = very satisfied – 1 = very unsatisfied

Do you currently get other income than income from working as a Wolt courier? Select all that apply to you. (Q4)

1. I don't get other income
2. I also get income from other contractor work
3. I also get income from a full-time employed job
4. I also get income from a part-time employed job
5. I also get student financial aid (opintotuki) from Kela
6. I also get housing allowance (asumistuki) from Kela
7. I also get basic social assistance (toimeentulotuki) from Kela
8. I also get unemployment benefits (työttömyysturva) from Kela
9. I also get other financial aid (sickness allowance, child benefits, other benefits from Kela, etc.)
10. I also get income from a source not listed above

As a rough estimate, how much of your total monthly net income do you get from delivering as a Wolt courier? (Q5)

1. 0–25 %
2. 26–50 %
3. 51–75 %
4. 76–100 %
5. I am not sure

Which of the following better describes your current work situation? (Q6)

1. It's my own choice to work as a Wolt courier
2. I work as a Wolt courier because I can't get any other work

Contractor or employee

Would you rather work as a Wolt courier as a contractor or as an employee? (Q7)

1. I’d prefer to continue working as a contractor
2. I’d prefer to work as an employee
3. It does not matter to me
4. I am not sure

(The following open-ended optional question is only one of the following and depends on the answer to 1 to 4 of the previous ones.)

Why would you prefer to work as a contractor? (Q7A)

Why would you prefer to work as an employee? (Q7B)

Why does it not matter to you? (Q7C)

Why are you not sure? (Q7D)

Below, please find two examples that describe how working as a Wolt courier as a contractor might differ from working as an employee. Please read the examples and then reflect on which option you’d prefer.

- *An example of working as a contractor:* You have higher gross income per delivery and per average hour, but you have to cover for e.g. your own pension, most insurances and sick leave, and you won’t have paid time off. You can affect your income by choosing when, where and how much you work. You can choose not to work, and you can take time off when you want. You don’t have a manager or a probation period. You can choose which delivery tasks you’ll do, and what vehicle (e.g. a bike or a car) you’ll use. You set your own goals. You can freely do other work.
- *An example of working as an employee:* You have lower gross income per delivery and per average hour, but your employer covers for e.g. your pension, insurances and sick leave, and you accrue paid time off. Your income is affected by your employer deciding when, where and how much you’ll work. You can’t choose not to work, and you can’t take time off when you want. You have a manager and a probation period. You have to do all the delivery tasks assigned to you, and the employer can require you to deliver with a certain vehicle (e.g. a bike or a car). Your employer sets your goals and monitors if you hit them. You can’t do other work as freely than as a contractor.

Would you rather work as a Wolt courier as a contractor or as an employee? (Q8)

1. I’d prefer to continue working as a contractor
2. I’d prefer to work as an employee
3. It does not matter to me
4. I am not sure

Contractor’s responsibilities, work as a courier & public discussion

How well do you think you know your responsibilities as a contractor – such as taking a self-employed person’s pension insurance (YEL) and paying taxes correctly? (Q9)
(can choose one of the options 1-5 + 6)

1. I know the responsibilities very well
2. I know the responsibilities well

3. I know the responsibilities on an average level
4. I know the responsibilities poorly
5. I don’t know the responsibilities at all
6. I use an invoicing service, an accountant, or some other help who takes care of the most important responsibilities for me

What do you think of the following claims? (Q10)
4 = I agree, 3 = I somewhat agree, 2 = I somewhat disagree, 1 = I disagree, I am not sure

1. The best things about being a Wolt courier are the freedom that comes with the work and the chance to affect my income
2. If someone else would tell me when, how much, and with which vehicle I need to deliver, I would consider quitting as a Wolt courier
3. One can get along financially with one’s net income as a Wolt courier
4. The freedom of being a contractor is more important to me than the security that comes with being an employee
5. Wolt is a fairer partner to couriers than Wolt’s competitors

What do you think of the following claims? (Q11)
4 = I agree, 3 = I somewhat agree, 2 = I somewhat disagree, 1 = I disagree, I am not sure

1. I might choose to deliver more if I notice I need more money
2. I might choose to deliver less if the weather is bad
3. I might choose to deliver less if I want to spend time with friends or family
4. I might choose to deliver less if I want to concentrate on other work, studies or other duties
5. I might choose to deliver a different amount on different days simply depending on how I feel
6. It is important to me that if I want, I can decline the delivery tasks offered to me

Have you followed the public discussion, e.g. in the media, about Wolt couriers? (Q12)

1. Yes
2. No
3. I am not sure

What do you think of the following claims regarding the public discussion about Wolt couriers? (Q13)
4 = I agree, 3 = I somewhat agree, 2 = I somewhat disagree, 1 = I disagree, I am not sure

1. The good sides of working as a Wolt courier have not come through enough
2. The bad sides of working as a Wolt courier have been exaggerated
3. The opinions of the majority of Wolt couriers have not come through enough
4. People seem to think that Wolt couriers don’t understand what’s best for them
5. In reality, Wolt is fairer towards its courier partners than what you might think based on the public discussion
6. The public discussion about Wolt couriers has been accurate and reflected the truth

Background

Age? (Q14)

16–17
18–24

25–34
35–44
45–54
55+

Highest education you have completed? (Q15)
Elementary school
Vocational school
High school
University of applied sciences
University (Bachelor’s or Master’s degree)
University (Doctor’s degree)
I have not attended any school

Which of the following describe your life situation? Select all that apply to you. (Q16)
I am a student
Being a Wolt courier is my main work
I do main work which is not being a Wolt courier
I do one form of part-time work that is not being a Wolt courier
I do several forms of part-time work that are not being a Wolt courier
I am unemployed

How long have you worked as a Wolt courier? (Q17)
0–3 months
4–6 months
7–12 months
12–24 months
25 months or more

How much do you estimate you deliver on a typical week? (Q18)
0 h
1–10 h
11–20 h
21–30 h
31–40 h
41+ h

Which of the following best describes how you have delivered with Wolt in the past three months? (Q19)
I have done Wolt deliveries as my main work
I have done Wolt deliveries on the side but regularly
I have done Wolt deliveries on the side every now and then
I have not done Wolt deliveries
